# Prognostic indicators in intensive care unit oncology patients: bridging short-term and long-term survival

**DOI:** 10.62675/2965-2774.20240262-en

**Published:** 2024-11-26

**Authors:** Fernanda Chohfi Atallah, Regis Goulart Rosa, Kathryn Puxty

**Affiliations:** 1 Universidade Federal de São Paulo São Paulo SP Brazil Universidade Federal de São Paulo - São Paulo (SP), Brazil.; 2 Hospital Moinhos de Vento Intensive Care Unit Porto Alegre RS Brazil Intensive Care Unit, Hospital Moinhos de Vento - Porto Alegre (RS), Brazil.; 3 Glasgow Royal Infirmary Intensive Care Unit Glasgow United Kingdom Intensive Care Unit, Glasgow Royal Infirmary - Glasgow, United Kingdom.

The number of cancer patients admitted to intensive care units (ICUs) has significantly increased over the past few decades. It is currently estimated that 20 to 30% of patients admitted to ICUs have oncological diseases. During this period, short-term survival rates for this patient population, including those with oncohematological conditions, have shown an increasing trend. This improvement can be attributed to advancements in several areas, including early diagnosis of organ dysfunction, major progress in cancer diagnosis, classification, and treatment, and more effective management of sepsis and respiratory failure, all of which contribute to the substantial positive clinical outcomes observed.^(1)^ However, decisions regarding the prognosis of critically ill cancer patients should go beyond considering only short-term survival. Long-term survival, quality of life and functional status are important outcomes, but an additional burden faced by ICU cancer survivors is the potential requirement to return to cancer treatment. While short-term critical care survival has improved, data addressing long-term outcomes after leaving the ICU remain limited.

In this issue of Critical Care Science, Silva et al. present the results of a retrospective cohort study of critically ill oncology patients with unplanned ICU admissions.^(2)^ The aim of this study was to identify the relative importance of clinical variables at ICU admission for short- and long-term mortality. This study was conducted from January 2017 to December 2018 at a specialized cancer center in Brazil and collected data on the cancer type (solid tumors, metastatic solid tumors, and hematological malignancies), the Eastern Cooperative Oncology Group (ECOG-PS) prior to hospital admission, the Sequential Organ Failure Assessment (SOFA) score, the Charlson Comorbidity Index, and mortality at 28, 90, and 360 days. A total of 3,592 patients were included, of whom 3,136 (87.3%) had solid tumors and 456 (12.7%) had hematological malignancies. Metastatic tumors were observed in 60.8% of the patients. The overall mortality rates at 28, 90, and 360 days were 33.3, 48.4, and 67.8%, respectively. Impaired functional status was associated with increased short-term mortality in all patients and with long-term mortality in patients with solid tumors. The type of cancer was more strongly associated with long-term mortality. In patients with hematological malignancies, the need for mechanical ventilation (MV) was the most important variable associated with increased short- and long-term mortality, followed by performance status. The SOFA score at admission was important for mortality prediction only in patients with metastatic solid tumors and oncohematological patients. The authors concluded that healthcare providers should consider performance status, the use of MV, and disease severity when discussing prognosis, goals of care, and end-of-life planning with patients and their families during the ICU stay.

This study is particularly important in that it specifies the factors related to short- and long-term mortality in oncology patients in an important cancer center in Brazil and aligns with other studies demonstrating that one of the greatest factors associated with poorer short- and long-term outcomes is preexisting performance status. For example, in a multicenter retrospective study analyzing factors associated with returning home after unplanned admission to the ICU among critically ill patients with metastatic solid malignancies, an ECOG-PS score ranging from 0 to 1 favored a 90-day return home, whereas malnutrition was independently associated with 1-year mortality.^(3)^ Additionally, in a multicenter observational study of patients with hematological malignancies admitted to seven Canadian ICUs between 2018 and 2020, only 21% of the entire cohort survived for 12 months.^(4)^ Notably, survival varied based on hematologic malignancy diagnosis and frailty status. Among those who were alive at 6 months, 25% continued with planned treatment, 23% had a modification to their treatment plan, and 39% were no longer candidates for treatment. The consistent association of impaired functional status with poor outcomes in cancer ICU patients across these studies highlights the necessity of considering baseline health status when determining the appropriateness of care, including decisions about ICU admission, organ support therapies, and end-of-life planning; additionally, it emphasizes the need for tailored post-ICU care focused on rehabilitation and preventing further health deterioration.

Another important finding from the study by Silva et al.^(2)^ is that the need for MV was the most significant factor associated with increased short- and long-term mortality in patients with hematological malignancies. However, the timing between ICU admission and the initiation of MV has not been reported. Additionally, data on the failure of noninvasive support were not available, and this variable may have influenced the outcomes observed in patients requiring MV. Failure of noninvasive ventilation leading to subsequent invasive MV has been demonstrated to increase short-term mortality;^(5)^ whether this is due to injurious effects of noninvasive support or the self-selection of a deteriorating patient cohort has not been delineated. Similarly, the poorer outcomes observed for the hematological patients who received MV in this study may not be causative but rather may be a marker of either the severity of acute insult or the physiological reserve.

Future studies should prioritize investigating the mediating effect of both acute care management strategies (e.g., MV, sedation, and early mobilization) and whether access to post-ICU physical, cognitive, and mental health rehabilitation can mitigate the impact of frailty on long-term mortality. Equally important is the assessment of other patient-centered long-term outcomes, such as health-related quality of life, return to cancer treatment, return to work or study, anxiety, depression, posttraumatic stress symptoms, and cognitive function, among critical care oncology survivors.

In conclusion, the study conducted by Silva et al.^(2)^ highlights the importance of clinical variables at ICU admission in predicting mortality in critically ill oncology patients. These findings reinforce the need for an individualized approach for managing these patients, considering not only immediate survival but also long-term outcomes.
