# Supplementary material for: Prognostic indicators in intensive care unit oncology patients: bridging short-term and long-term survival
Source: Crit Care Sci. 2024 Nov 26;36:e20240262en. doi: 10.62675/2965-2774.20240262-en (PMC11634240; doi:10.62675/2965-2774.20240262-en)
Supplement: Translation

# Translation: Indicadores prognósticos de pacientes oncológicos em unidades de terapia intensiva: conectando a sobrevida em curto e longo prazo

**DOI:** 10.62675/2965-2774.20240262-pt

**Authors:** Fernanda Chohfi Atallah, Regis Goulart Rosa, Kathryn Puxty

**Affiliations:** 1 São Paulo SP Brasil Universidade Federal de São Paulo - São Paulo (SP), Brasil.; 2 Porto Alegre RS Brasil Unidade de Terapia Intensiva, Hospital Moinhos de Vento - Porto Alegre (RS), Brasil.; 3 Glasgow United Kingdom Intensive Care Unit, Glasgow Royal Infirmary - Glasgow, United Kingdom.

O número de pacientes com câncer internados em unidades de terapia intensiva (UTIs) aumentou significativamente nas últimas décadas. Atualmente estima-se que 20 a 30% dos pacientes internados em UTIs tenham doença oncológica. Durante esse período, a sobrevida a curto prazo dessa população, incluindo aqueles com doença onco-hematológica, vem aumentando. Essa melhora pode ser atribuída a avanços em diversas áreas, incluindo identificação precoce de disfunção orgânica, progressos no diagnóstico, classificação e tratamento do câncer, e otimização no manejo da sepse e da insuficiência respiratória.^(1)^ No entanto, as decisões relativas ao prognóstico de pacientes críticos com câncer devem ir além da sobrevida a curto prazo. A sobrevida a longo prazo, a qualidade de vida e o estado funcional são desfechos importantes, mas um fardo adicional enfrentado pelos pacientes oncológicos que tiveram alta da UTI é a possibilidade de retornar ao tratamento oncológico. Embora a sobrevida a curto prazo em pacientes críticos tenha aumentado, ainda são limitados os dados que abordam os desfechos a longo prazo após alta da UTI.

Nesta edição da *Critical Care Science*, Silva et al. apresentam os resultados de um estudo de coorte retrospectivo em pacientes oncológicos críticos com internação não planejada na UTI.^(2)^ O objetivo desse estudo foi identificar a importância das variáveis clínicas na admissão na UTI quanto à mortalidade a curto e longo prazo. Este estudo foi realizado entre janeiro de 2017 e dezembro de 2018 em um centro especializado em câncer no Brasil e coletou dados do tipo de neoplasia (tumores sólidos, tumores sólidos metastáticos e malignidades hematológicas), da escala de desempenho do *Eastern Cooperative Oncology Group* (ECOG-PS) antes da admissão hospitalar, da pontuação do *Sequential Organ Failure Assessment* (SOFA), do Índice de Comorbidade de Charlson e da taxa de mortalidade em 28, 90 e 360 dias. Foram incluídos 3.592 pacientes, dos quais 3.136 (87,3%) tinham tumores sólidos e 456 (12,7%) tinham malignidades hematológicas. Tumores metastáticos foram observados em 60,8% dos pacientes. As taxas de mortalidade geral em 28, 90 e 360 dias foram de 33,3, 48,4 e 67,8% respectivamente. O estado funcional prejudicado foi associado ao aumento da mortalidade a curto prazo em todos os pacientes e ao aumento da mortalidade a longo prazo em pacientes com tumores sólidos. O tipo de câncer foi mais associado à mortalidade a longo prazo. Em pacientes com neoplasias hematológicas, a necessidade de ventilação mecânica (VM) foi a variável mais importante associada ao aumento da mortalidade a curto e longo prazo, seguida pela escala de desempenho. O SOFA na admissão foi importante para prever a mortalidade somente em pacientes com tumores sólidos metastáticos e em pacientes onco-hematológicos. Os autores concluíram que os profissionais de saúde devem considerar a escala de desempenho, o uso de VM e a gravidade da doença ao discutir o prognóstico, as metas de cuidado e o planejamento de fim de vida com os pacientes e seus familiares durante a internação na UTI.

Este estudo é importante pois especifica os fatores relacionados à mortalidade a curto e longo prazo de pacientes oncológicos em um grande centro no Brasil e se alinha a outros estudos que demonstram que um dos maiores fatores associados a desfechos desfavoráveis a curto e longo prazo é a escala de desempenho preexistente. Em um estudo retrospectivo multicêntrico que analisou os fatores associados ao retorno para casa após admissão não planejada na UTI entre pacientes críticos com neoplasias sólidas metastáticas, uma pontuação ECOG-PS de zero a um favoreceu o retorno para casa em 90 dias, enquanto a desnutrição foi independentemente associada à mortalidade em 1 ano.^(3)^ Em um outro estudo observacional multicêntrico de pacientes com neoplasias hematológicas admitidos em 7 UTIs canadenses entre 2018 e 2020, apenas 21% de toda a coorte sobreviveu por 12 meses.^(4)^ Notavelmente, a sobrevida variou em função do diagnóstico de neoplasia hematológica e do grau de fragilidade. Entre aqueles que estavam vivos aos 6 meses, 25% continuaram com o tratamento planejado, 23% tiveram uma modificação no plano de tratamento e 39% não eram mais candidatos ao tratamento. A associação consistente do estado funcional prejudicado com desfechos desfavoráveis em pacientes com câncer na UTI nesses estudos destaca a necessidade de considerar o estado de saúde basal ao determinar a adequação do cuidado, incluindo decisões sobre a admissão na UTI, terapias de suporte a órgãos e planejamento de fim de vida; além disso, enfatiza a necessidade de um atendimento individualizado pós-UTI voltado à reabilitação e à prevenção de maior deterioração da saúde.

Outro achado importante do estudo de Silva et al.^(2)^ é que a necessidade de VM foi o fator mais associado ao aumento da mortalidade a curto e longo prazo em pacientes com neoplasias hematológicas. Entretanto, o tempo entre a admissão na UTI e o início da VM não foi descrito. Também não havia dados disponíveis sobre a falha do suporte ventilatório não invasivo, e essa variável poderia ter influenciado os desfechos observados em pacientes que necessitaram de VM. É demonstrado que a falha da ventilação não invasiva que leva à VM invasiva subsequente aumenta a mortalidade a curto prazo.^(5)^ Ainda não foi estabelecido se este fato se deve aos efeitos deletérios do suporte não invasivo ou à seleção de uma coorte de pacientes em deterioração. Da mesma forma, os desfechos desfavoráveis observados nos pacientes hematológicos que necessitaram de VM neste estudo podem não ser causais, mas um marcador da gravidade do insulto agudo ou da reserva fisiológica.

Estudos futuros devem priorizar a investigação do efeito mediador das estratégias de manejo de cuidados intensivos (por exemplo: VM, sedação e mobilização precoce) e se o acesso à reabilitação física, cognitiva e de saúde mental pós-UTI pode atenuar o impacto da fragilidade na mortalidade a longo prazo. Igualmente importante é a avaliação de outros desfechos a longo prazo centrados no paciente, como qualidade de vida relacionada à saúde, retorno ao tratamento oncológico, retorno ao trabalho ou aos estudos, ansiedade, depressão, sintomas de estresse pós-traumático e função cognitiva, entre os pacientes oncológicos sobreviventes após internação na UTI.

Em conclusão, o estudo realizado por Silva et al.^(2)^ destaca a importância das variáveis clínicas na admissão na UTI em prever a mortalidade em pacientes oncológicos. Esses achados reforçam a necessidade de uma abordagem individualizada no manejo desses pacientes, considerando não apenas a sobrevida imediata, mas também os desfechos a longo prazo.
